# Implementation of eMental health technologies for informal caregivers: A multiple case study

**DOI:** 10.3389/fdgth.2023.1130866

**Published:** 2023-03-24

**Authors:** Sofia Bastoni, Lisette van Gemert-Pijnen, Robbert Sanderman, Anne van Dongen

**Affiliations:** ^1^ Department of Psychology, Health and Technology, Centre for eHealth and Wellbeing Research, University of Twente, Enschede, Netherlands; ^2^ Department of Health Psychology, University Medical Center Groningen, Groningen, Netherlands

**Keywords:** eHealth, implementation, informal care, eMental health, CFIR, business modeling, values, stakeholder involvement

## Abstract

**Introduction:**

Informal caregivers offer continuous unpaid support to loved ones who are unable to live independently. Providing care can be a very burdensome commitment, that heavily impacts informal caregivers’ mental health. eMental health is a possible, yet challenging, solution to improve caregivers’ mental health and their overall experience of caregiving. In fact, eMental health technologies often face challenges of implementation. The present work gathers knowledge on how to best deal with these challenges by collecting testimonies of implementation experts of eight eMental health technologies for informal caregivers with the aim of comparing them and extracting lessons learned.

**Methods:**

For this multiple case study, technologies were selected (through informal suggestions and independent search) according to the following inclusion criteria: they were intended for informal caregivers as main user group, were aimed at improving informal caregivers’ mental wellbeing and caregiving experience and were available and running in real life settings in Europe. Ten interviews were conducted (two pilots and eight included cases). The interviewees were asked to provide a description of the technology and its aims and their implementation approach, method and frameworks used. Finally, determinants of implementation, the influence of the Covid-19 pandemic on implementation processes and lessons learned were investigated.

**Results:**

The results highlight key differences between technologies developed within academia and the industry regarding efficacy testing and use and use and choice of frameworks. Also, similarities in terms of recognized barriers such as financing are illustrated.

**Discussion:**

Possible ways to overcome main barriers and examples of best practices, such as structuring a business model and discussing tool maintenance and long-term hosting in advance, are discussed.

## 1. Introduction

Informal caregivers are spouses, adult children, family members, friends, and neighbors who provide care to a chronically ill, frail, or otherwise unable to live independently loved one (1). Informal caregivers offer continuous unpaid help in different daily tasks like shopping, grooming, household chores and administrative duties and further often provide emotional support and assistance with medical treatments (1, 2). Informal care is a very prominent phenomenon. In fact, it is estimated that 17% of the population in Europe is made up by informal caregivers (3) and in the Netherlands alone, over six million provide unpaid care to a loved one (4).

Caregiving can be a very burdensome and intense commitment (5), which can dramatically impact the wellbeing, physical and mental health of caregivers (6, 7). Even higher levels of caregiver burden and intensity were reported during the SARS-CoV-2 pandemic. Those were often related to the governmental restrictions imposed to stop the spread of the virus, since isolation can exacerbate the detrimental consequences of caregiving (8). Another consequence of governmental restrictions and isolation was the dramatic increase in usage of internet services (9), which opened new possibilities for caregiver support, both informal (contact with friends, leisure activities etc.) and formal (such as therapy, support groups etc.).

The use of technology for mental health is considered a promising solution to expand the access and delivery of care (10), by addressing physical and geographical barriers. Beyond being available and easily accessible, there is evidence that eMental health technologies have positive impacts on caregivers’ wellbeing by effectively improving their knowledge and self-efficacy and reducing depression and anxiety symptoms (11, 12).

eHealth can be defined as the use of technology to improve health, wellbeing, and healthcare (13). Although eHealth is a promising solution to increase access to care (14, 15), innovations often face challenges of implementation and upscaling, ending up abandoned despite being proven effective (16). Since literature addressing this phenomenon is abundant (10, 17, 18), the present work focuses on a specific subset of the problem: the implementation of eMental health technologies for informal caregivers.

Implementation can be defined as efforts to adopt, upscale and integrate eHealth technologies into healthcare systems (13). Ideally, successful implementation leads to desirable outputs such as the acceptance of the technology among its stakeholders, a high degree of fidelity between how a technology is implemented and how it was originally intended by its developers, and its sustainability on the long term (19). To provide guidance toward successfully implementing eHealth technologies, many guidelines and frameworks have been developed, such as the as (i) the Non Adoption, Abandonment, Scale-Up, Spread and Sustainability (NASSS) Framework (16), (ii) the CeHReS Roadmap (13), (iii) the Consolidated Framework for Implementation Research (20). The CFIR and the NASSS Framework focus on respectively identifying and explaining the factors influencing implementation and assessing the level of complexity in the interplay of these factors. The CeHRes Roadmap, on the other hand, offers a step-by-step guideline for implementing eHealth into healthcare system. While these frameworks have different aims, it is possible to recognize a common ground in the principal domains influencing implementation of eHealth, and that is the interaction between a technology, its users (and stakeholders) and broader contextual elements.

Although useful tools and guidelines are in place, implementation is often seen as separate from and a consequence of design (13, 21), resulting in implementation failures and abandonment. In fact, a recent umbrella review (22) found that implementation-related information is often underrepresented in review papers describing eHealth technologies for informal caregivers, and guidance on effort optimization to integrate innovations in the wider healthcare systems is still lacking. An in-depth analysis of eMental health technologies that are available in real life settings was conducted, with the aim of collecting testimonies of implementation best practices in the domain of technologies to support informal caregivers’ mental wellbeing.

Specifically, cases of eMental Health technologies for informal caregivers are described and compared, focusing on (i) implementation approaches and methods, (ii) attitudes toward and experiences with implementation frameworks and theories, (iii) perceptions of implementation barriers and determinants and (iv) elements of business modeling.

## 2. Methods

This multiple case study is made up by (i) individual online interviews with implementation experts in eMental health technologies for informal caregivers and (ii) analyses of additional materials [e.g., Websites, (grey) literature, business models etc.]. The experts were recruited through snowball and purposive sampling in different European countries. The project received ethical approval from the University of Twente (Request number 220018). Prior to the interviews, participants signed an informed consent form.

### 2.1. Sample

#### 2.1.1. Step 1: selecting cases of eMental health interventions for informal caregivers

The technologies included in the present case study were selected through a combination of informants’ suggestion (recruited with convenience and snowball sampling) and independent search by the researchers (through academic literature and internet search). Specifically, (non) academic professionals related to eHealth and informal caregivers were recruited through social media (Facebook), direct email, and through presentations at international conferences. Using a single question survey, the informants were asked to indicate examples of eMental health technologies supporting informal caregivers they knew of or used. The informants were able and encouraged to spread the survey within their network contacts. In total, 27 technologies were suggested or identified through independent search. Of those 27, 10 were excluded because they did not meet the inclusion criteria. Contact people for each of the 17 technologies remained were identified and approached. Of those 17 technologies, 7 were excluded because (a) it became clear that the technology did not meet the inclusion criteria after having a first discussion with the contact person, (b) the technologies were going to be discontinued soon thereafter or (c) the approached contact person did not respond to our request. In the end, 2 technologies were included as pilots, and 8 were included as cases.

#### 2.1.2. Step 2: technology and participant inclusion

Suggested technologies that met the following criteria were included: (i) they were intended for informal caregivers as main user group, (ii) were aimed at improving informal caregivers’ mental wellbeing and caregiving experience and (iii) were available and running in real life settings in Europe. Technologies in design or evaluation stages, and technologies that were intended for the general public or patients (as opposed to their caregivers) were excluded. Once the cases were identified, relevant contact persons were approached and asked to be interviewed. More specifically, people who were involved in or knowledgeable about the implementation process of the included technologies were considered for inclusion. Job descriptions of the interviewees vary depending on the type of company or organization: implementation managers, researchers, product managers and policy managers were involved.

### 2.2. Procedure

Semi-structured interviews took place online for an approximate duration of 1 h. Two pilot interviews were conducted to test the interview guide in different countries. Interviews were recorded and transcribed using AmberScript. The transcripts were sent to the interviewees for them to be able to check or retract information, but no changes were made to the original verbatim. Interview data was analyzed and coded by one researcher (SB) and checked by a second researcher (AD) using the software package Atlas.ti 9. Discrepancies were solved by consensus within the group of authors. First, relevant quotes were selected and categorized deductively based on the interview guide (see paragraph 2.3 Analytical Framework). Subsequently, selected quotes were further categorized inductively into overarching codes described in detail in the result section.

### 2.3. Analytical framework

The interview guide was informed by insights from prior research (22) conducted within the team and overarching themes from aforementioned implementation frameworks and theories i.e., (i) the Non Adoption, Abandonment, Scale-Up, Spread and Sustainability (NASSS) Framework (16), (ii) the CeHRes Roadmap (13), (iii) the Consolidated Framework for Implementation Research (21). Overlapping determinants of implementation identified in these frameworks are the characteristics of the technology itself; the characteristics of the end-users and contextual elements, both referring to the wider context (e.g., the healthcare system) and inner context (e.g., organizational culture).

The interview was made up of four main areas. Initially, general questions were asked with the aim of achieving a description of the technology and its aims. Later, more specific questions on implementation approaches, methods and frameworks were asked, together with barriers and determinants of implementation and the influence of the Covid-19 pandemic on implementation processes. The complete interview guide can be found in Supplementary Material.

When available, additional information was analyzed to enrich or expand on the interviews. For example, interviewees at times referred to additional materials (e.g., grey literature and scientific publications on the companies). Moreover, the business model of the different technologies was co-created together with the interviewees as a follow-up. Practically, interviewees were called to comment on their business models during the interviews, using the Business Model Canvas (23) to structure the information. The results will focus on three business models domains: financial aspects, stakeholder involvement and value proposition. Value proposition refers to elements which were considered essential in developing the technologies, as values can be defined as what is important for a certain group of people (24).

## 3. Results

Eight eHealth technologies launched and running in 5 European countries were included (Italy, the Netherlands, United Kingdom, Belgium and Germany). Most technologies are eMental health digital programs (also referred as “modules”), available on websites or platforms (such as Minddistrict) with exercises, information, and resources for informal caregivers. An online intervention, an adaptive website and a digital calendar are also included.

Two main types of innovations were identified, and their distinction will serve as a base for comparison in the present work. Namely, the present case study compares technologies with academic origins and technologies developed within the industry context. More specifically, technologies developed within the academic context are usually the result of academic research projects funded by grants. On the other hand, technologies developed within the industry are usually products developed by business-to-business companies (B2B) and licensed to healthcare organizations.

At the beginning of this section each case is briefly described with an emphasis on the content level, theoretical background (when applicable), key elements of the implementation strategy employed and quotes from interviews to provide an example. In the following section, more detailed results are laid out, describing the specific approach towards implementation for each technology and the corresponding business models, focusing on stakeholder involvement and value proposition. Lastly, a brief overview of the main overarching themes, extracted lessons learned, and the effect of the Covid-19 pandemic on implementation practices, are described.

### 3.1. Overview of codes

In total, 49 codes were generated and later grouped into 6 categories, according to the identified overarching themes. The codes can be grouped as follows: **(1) Descriptive codes**: descriptive information on the eHealth technology from a hardware, software, and content point of view. Other elements such as its features, ethical considerations, and needs assessment process are included. The results from these codes are described in the next paragraph, providing an overview of the included cases. **(2) Implementation codes:** all elements regarding the implementation theories, processes, and experiences within the selected technologies. Information about (i) barriers (general, specific, or recurring), (ii) other determinants (e.g., the influence of the Covid-19 pandemic), (iii) efficacy or other types of testing, (iv) experiences with existing frameworks of implementation, (v) tool maintenance or updates, (vi) stages of implementation are included. **(3) Business model:** encompasses information on the existence, sophistication, description, and stage of business models within the included cases. **(4) Values:** This family of codes considers both psychological values (e.g., those which are related to the wellbeing of the caregiver and theory driven psychological aspects) and practical values (e.g., privacy, security, value proposition). **(5) Stakeholder involvement:** collaborations with external parties, end-users, and all elements regarding stakeholder involvement. **(6) Lessons learned:** direct experience testimonies that provide lessons learned. An overview of the codes and brief definitions are provided in Supplementary Material.

#### 3.1.1. Pilot interviews

Pilot interviews were used to calibrate the final interview guide. For this reason, pilot interviews will not be described as self-standing cases. However, interesting learning points from these interviews will be integrated in the result and discussion sections.

##### 3.1.1.1. Friend functionality—minddistrict

Minddistrict is a healthcare company active in the Netherlands, the United Kingdom and Germany. The Minddistrict platform offers a number of digital programs (or modules) developed within the company, but also offers a distribution platform for modules developed elsewhere (see Case 4). The pilot interview revolved around a specific functionality of the platform. A separate interview focusing on a specific caregiver module was conducted (Case 3). The “Friend Functionality” allows family members or friends to take part in the care process by offering them access to a self-care module and allowing them to take part in (some) conversations between the patient (their loved one) and therapist. The functionality was designed consequently to direct request from end-users. Most interestingly, the interviewee states that from their perspective, holistic involvement of the caregiver in the care process is not common practice, however it is considered important.

“*From my perspective, I think including informal caregivers is not that common yet, even though everybody talks about it and then including them via E health is even a step further from happening already so I think we have some steps to take there to really make this happen*.”

##### 3.1.1.2. A casa ma non da soli (lit. at home, but not alone)

“A casa ma non da soli” is the online adaptation of a series of seminars developed for caregivers and care recipients. The seminar was created by an Italian research institute (Istituto di Ricerca a Carattere Scientifico -INRCA) and licensed to local caregiver organizations with a “train-the-trainers” business model. It was delivered free of costs to the users. Because of the pandemic, the institute decided to try out an online edition which yielded mixed results. On one hand, it helped addressing geographical barriers, inequalities in access to health and the need for caregiver support which was no longer available during the pandemic. However, the group cohesion was hindered by the online mode. Finally, the technology struggled with reaching a large proportion of the target group older adults, the elderly, given their proclivity towards low digital literacy, which represented a barrier. Due to pandemic circumstances, the adaptation had to happen quickly and, although a thorough implementation plan had not been developed; the interviewee recognized that was part of the reason why the project was not adopted in following editions.

“*We didn't have a really implementation theory and this is a pity, but I understood that later on… And yeah, we just think about that and then have a lot of meetings to do reasoning and share a lot of thinking about that with these professionals. But we didn't we didn't have a proper implementation plan or proper implementation theory, you know, it was not it… It isn't still our main area of competence*.”

#### 3.1.2. Included cases (descriptive codes)

Case 1: Partner In Balans

Partner In Balans (PiB) is an online intervention for people with dementia (PwD) and their caregivers. It is based on the principals of self-management and helps informal caregivers of PwD adjusting to their role. On a content level, the website revolves around self-management, positivity and teaching caregivers a method to cope instead of just skills. Addressing the divide between real world settings and academia, the interviewee highlights that technology often faces a tradeoff between getting more stability of cashflow in the industry setting but having to give up being able to do research on it. PiB's implementation strategy, planning, and business modeling are described in detail in another publication (25).

“*This has been a successful implementation. It could run itself kind of thing. On the other hand, it is a great tool to do research with and find out caregivers needs, so we can make more and more modules like the way it exists now. It wouldn't be possible to do it without research grants. It would at a certain point sort of stagnate and not be up to date anymore […] So you could make the argument that the module is basically where it needs to be, but I guess if you're talking about dream scenarios, you would want it to be offered by a health insurer or something, and it would be like freely accessible to everyone and the health insurer would compensate for it. But then, of course, in that scenario, we can't do research on it anymore. I think it would be sort of set in stone*.”

Case 2: Mantelzorg Balans (Caregiver Balance)

Mantelzorg Balans is an adaptable website for informal caregivers of palliative care patients. The website has three main functions: it provides reliable sources of information regarding topics of interest for informal caregivers, exercises promoting a balanced practice of the caregiving role, and an open field function to write down memories and store them. The website's theoretical background is the Acceptance and Commitment Theory (26). In terms of content, the website deals with emotions, goals, and wishes. It was developed in the context of a research grant, addressing the imbalance of tools available for caregivers (compared to the ones aimed at patients), and in general the lack of active support (as opposed to reactive) for informal caregivers. The technology was not tested for effectiveness; however, a small round of pilots, co-design, usability testing, and a survey evaluation were carried out. The tool will remain available to end-users 5 years after launch because of premade agreements with funding organizations on the grant. The interviewee reported using an existing implementation framework (CeHRes Roadmap) (13) but described difficulty translating the framework into a practical guideline (see also Table 1).

**Table 1:** Experiences with implementation theories, models, and frameworks.

eMental health technology	Origin	Implementation framework	Quote
Case 1: Partner In Balans	Academia	Consolidated Framework for Implementation Research (21) and Business Model Canvas. Formerly they used the Medical Research Council Framework for Complex Intervention (27)	“I went back and forth between all the frameworks so many times. There's so many of them, and they are so similar. But you’re still afraid of choosing the wrong one somehow.”
Case 2: Mantelzorg Balans	Academia	CeHRes Roadmap (13)	“I found it a bit difficult to apply in the real world”
Case 3: Terug in balans (voor naasten)	Industry	They eventually developed their own implementation method. The Intervention Mapping approach (28) was also mentioned as a reference	“Yes, and that's a framework that we made ourselves, and it was developed also with an external company who helped us with this. And it's like years of experience combined with their guidance and then an implementation method was born. And over the years we've adapted it”
Case 4: Goed Ernaast	Industry	Did not follow a specific implementation theory, framework, or approach.	“When we first launched the module, we did not have as good knowledge of implementation as we have now, so we just launched it and distributed also on Minddistrict”
Case5: Mantelzorg	Industry	Joined Impact Plan https://winningbydesign.com/resources/blueprints/create-a-joint-impact-plan/ (29)	“I feel that frameworks overlap so much, and they all look at different kinds of domains or levels”
Case 6: Me-We	Academia	Did not follow a specific implementation theory, framework, or approach.	“The implementation elements and steps we considered are bottom up, meaning they come from direct experience. But we did not refer to an existing model”
Case 7: Anziani e non solo—lit: not only Elderly (eLearning platform)	Industry	Did not follow a specific implementation theory, framework, or approach.	“We don't rely on any specific model. Planning starts from the very beginning, when the product is not there yet.”
Case 8: Luna	Industry	Critical success factors, indicated by the Knowledge Center for the Dutch Healthcare Market) https://kenniscentrumzorg.com/ (30) Crossing the chasm (31) was also mentioned as an additional reference.	“Within the critical success factors for implementing care technologies is quantifying the value. So we knew that it was really important, and who is going to determine if the value is enough to finance the project is the insurer”

“*We used the CeHRes road map, but somehow it was a bit difficult for me to apply it in the real world. […] That's the also the part that you write in the grant agreement, you say hey, we're going to do interviews with informal caregivers. We're going to do interviews with patients and we're going to do interviews with care professionals. Then we will have a focus group. And then you talk about values and then you have the design phase in which you do co-design and then you also have a business modeling part in it. So that's how you write it up and how you foresee it. But the practice is in my opinion a bit different. And it's also it has to do with my inexperience*.”

Case 3: Terug in balans (voor naasten)—lit: “back in balance (for loved ones)”

Terug in balans (voor naasten) is a self-help module for informal caregivers, also developed by Minddistrict. On a content level, the module aims to educate caregivers through exercises, videos, and text fragments about caregiver burden and the importance of keeping a balance between personal life and caregiver duties. The interviewee reported that they used to follow an existing implementation framework. Together with an external partner they then ended up designing their own blueprint, informed by practice and existing literature. The blueprint is continuously updated according to new insight, adaptable to their clients’ needs, and is based on setting and measuring goals for the implementing organization, as well as providing practical and technical support.

“*Yes, that's a framework that we made ourselves […] and it was developed also with an external company who helped us with this, and it's like years of experience combined with their guidance and then an implementation method was born. And over the years we've adapted it*.”

Case 4: Goed Ernaast (nonliteral: good neighbor)

Transfore is a mental healthcare organization for forensic patients. Their portfolio includes a module specifically dedicated to caregivers, namely “Goed Ernaast”. Its content aims at enabling and improving caregivers’ feeling of agency in the care process. Further, it includes information about burnout awareness and prevention as well as tips to provide better care. Similarly to Minddistrict's experience with the friend functionality described in the pilot case, end-users of Transfore's modules reached out reporting a need for support. Specifically, caregivers of forensic patients reported feeling stigmatized or blamed for having relatives in forensic mental health care. The module was not tested for efficacy, but only a small pilot was run on the general population. In fact, it is difficult for the company to reach caregivers of forensic patients for anonymity reasons. Another similarity to Minddistrict is found in the fact that caregivers are not Transfore's primary target group (patients are). They do not report following a specific implementation plan, but rather try to make the product as visible as possible by sharing it for free on other platforms and keeping in mind their blended care vision.

“*We just put it on Minddistrict and we gave some exposure to the module and on the website of Transfore. In intakes sometimes the informal caregiver came with our patient and we said: “well, there is a module”. Actually, that's what we did at that moment, that was the whole implementation process […] that's what we did and what we thought we could do at the moment*.”

Case 5: Mantelzorg (lit. informal caregiving)

Mantelzorg is a eMental health module for informal caregivers, developed by Therapieland. On a content level, the module focuses on burden prevention and separating the end-users’ identity from their caregiving duties. The module was developed following explicit manifestation of the need for this module from the side of an end-users. The module was not tested for efficacy, but it was co-designed with experts-by-experience. The interviewee reports the company using the Joint Impact Plan (29) as an implementation framework, and valued the fact that it is simple, brief, agile and highly customizable (see also Table 1).

“*When we just started, we would just read it into literature. You have so many interesting things on implementing that… we were just trying to be thorough, but the result was that we had an implementation plan of 15 to 20 pages. It's not something you want to work with. It's we make this very nice. It ends up in the shelf and you don't use it anymore and the joint plan makes sure that it's short, it's the basic of what you need*.”

Case 6: Me-we

Me-We is an app aimed at young caregivers developed in the context of a academic research project. It was originally designed for blended use, together with sessions of group counseling, but can still be used as a standalone. The group activities were originally planned as face-to-face but took place online because of the social isolation measures. The intervention is based on the DNA-V model (32) and helps teen caregivers reflect on their own set of values and promotes caregiver resilience. The intervention was tested for efficacy with a randomized controlled trial (RCT) in different countries (33). For this case study, the interview focused on the Italian adaptation. The interviewee recognized main barriers to implementation involved data ownership, because of the international context, as well as ethical consideration working with minors. Although the project ended, arrangements were made to continue host and run the app on app stores making it still downloadable and usable. A business model was not designed and, to allow the content to be free for users, maintenance arrangements with funding organizations were made.

“*The app was developed by our Swedish partners, who made the commitment to maintain the app on the app stores. We have also found a pretty functional way to maintain the tool, and every country can autonomously manage users and update contents. So, the app is fully available still, although the project is over. On the other hand, us- and the other partners as well- are trying to find funds to repeat the intervention, regardless of heavy bureaucracy*.”

Case 7: Anziani e non solo—lit: not only Elderly (eLearning platform)

Anziani e non solo is a nonprofit organization for informal caregiving active in Italy that has existed for nearly 20 years and focuses on conducting research, development, and education. Among their different activities, Anziani e non solo designed an e-learning platform for formal and informal caregivers. The online modules are self-paced and have video lessons, exercises to monitor progress and downloadable materials. Some of the courses were developed in academic contexts (i.e., through research grants) and remained available on the platform, while others were developed internally for exclusively internal use. The vision behind the creation of the platform was addressing geographical and access barriers to education on caregiving for caregivers. The courses vary in content: some are disease specific, others have a more social focus, but all of them are developed under the theoretical umbrella of psychoeducation (34). Courses are accessible for users by credit card purchase or *via* their companies’ welfare. In fact, the courses are often licensed to companies who offer them to their employees as benefits. The modules are not tested for efficacy, and they do not follow an existing implementation framework; however, they try to keep aspects of implementation in mind from the first stages in design.

“*We don't rely on any specific model. Planning starts from the very beginning, when the product is not there yet. We planned according to time, mode and resources, until dissemination. Then of course the plans are updated, but we do start planning from the very start of the design until distribution*.”

Case 8: Nedap-Luna

Luna is a digital day calendar for people with cognitive problems that was originally construed within a master thesis project and was eventually developed by the Dutch company Nedap. It consists of an eReader screen with durable battery autonomy. Luna is connected to an integrated care platform, that enables caregivers to enter information on the calendar, which then become visible to the users. Through that, informal caregivers help their loved ones with day structure and memory, alleviating their worries, caregiving time and visits. They recognize financing as the biggest barrier to implementation and see their main implementation goal as to help their clients quantify the added value of adopting Luna. To do so, they try to measure the impact Luna has on caregivers’ time and efforts.

“*We get in touch with the caregivers to do baseline measurements on what care time spent at the client, what kind of care activities they do at the client and on which one Luna is going to support them with. And then we evaluate after one month, three months and five months to see what kind of care time is taken off these measurements with Luna's support*.”

### 3.2. Implementation approach

This family of codes gives an overview of implementation theories, practices and approaches that emerged in the interviews. Specifically, experiences with frameworks, barriers, determinants, and information regarding the stage of implementation of the included cases is provided. Information about specific aspects of implementation such as values and value proposition, business model and stakeholder involvement are detailed in the following paragraphs. Table 1 describes the theories, frameworks and approaches that implementation experts used, as well as some relevant quotes.

Privacy and data safety matters were mentioned by different interviewees as implementation barriers. Relatedly, data ownership is also an obstacle, especially when minors are involved or when dealing with different GDPR regulations. Moreover, in the case of technologies that were developed in the industry setting, developing companies are often not owners of usage data. For this reason, it is not an option for them to conduct systematic effectiveness evaluations. In fact, although all technologies seem to have deep roots in scientific literature (content-wise), all technologies developed within the industry and most of the technologies developed in academia are not evidence based. However, in most cases some qualitative testing (e.g., small pilots with the target group or co-design methods) were conducted.

Other barriers relate to the resistance to change from relevant stakeholders’ perspective. For example, those technologies that are used alongside face-to-face therapy are highly impacted by the therapists’ and coaches’ attitude toward the technology. Coaches, trainers and other figures involved with the delivery of the technologies act, according to the interviewed experts, as human barriers or facilitators, depending on their attitude. Similarly, technology literacy from the end-users’ side also acts as a barrier. Other barriers are more circumstantial. For example, within the Italian included cases, a barrier often comes from the public healthcare system, which does not provide reimbursement solutions (e.g., *via* insurance reimbursements) to end-users for eMental health products. In this sense, it is necessary to make different arrangements when building a business model, in case end-users are not meant to be the people paying for the service being delivered.

### 3.3. Business models

Business models are a set of choices made by companies to create and deliver value to their customers and are used to analyze their viability (13). In this sense, knowing how technologies are funded and what kind of business model they hold, is crucial to understand their implementation challenges and facilitators, as well as to be able to make comparisons between different technologies.

As already mentioned, all included cases are technologies available to use in real life settings. However, their level of implementation and self-sustainability is variable. Based on their responses, Table 2 synthesizes their business model and differentiates by end-users and clients (providing financing). Generally, insurance-based technologies and subscription-based technologies can be considered as part of a self-sustaining implementation, while the ones who are dependent on research grants have a more uncertain outcome in the long term.

**Table 2:** Business models of included cases.

Case	Financing	Client	End-users
Partner In Balans	Research grants	Municipality	Informal Caregivers of people with Dementia
Mantelzorg Balans	Research grants	-	Informal Caregivers of palliative patients
Terug in balans (voor naasten)	Insurance based	Mental Health Care Organizations	Informal Caregivers of people who are in blended therapy
Goed Ernaast	Insurance based	-	Informal Caregivers of forensic mental health patients
Mantelzorg	Insurance based	Mental Health Care Organizations	Informal Caregivers of people who are in blended therapy
Me-We	Research grants	-	Young Informal Caregivers Caregivers (teenagers)
Anziani e non solo, eLearning platform	Subscription	(In)formal Caregivers of elderly people	(In)formal Caregivers of elderly people
Luna	Insurance based	Healthcare organizations	Informal Caregivers of people with cognitive syndromes/disabilities

### 3.4. Stakeholder involvement

Stakeholders are all relevant individuals and/or companies that share an interest in the added values of a technology. A widely accepted definition of stakeholders is “any group or individual who can affect or is affected by” (35) in this case, eMental health technologies. The current paragraph elaborates on the group of codes “stakeholder involvement”, mainly addressing the different stakeholder groups considered in the included cases and their role in the implementation.

One of the most frequently mentioned stakeholders are technology companies. Most often, for technologies which have their origin in academic research, the technical part of design is outsourced. In this case, stakeholders are providers of a service on demand. Similarly, other types of experts were involved as stakeholders to provide their consultation. Namely, healthcare professionals were involved to ensure the accuracy of contents within the modules, apps or websites, and experts from academia were involved in order to offer guidance in research activities.

All interviewed experts mentioned end-users (in this context informal caregivers) as their primary stakeholder group. Most implementation experts within the included technologies declared having a sort of co-design approach with their end-users, but with different extents of involvement. One of the first steps of end-users’ involvement is commonly undertaking a needs assessment, using mainly interviews, focus groups and questionnaires. Organizations like Transfore and Minddistrict reported creating the caregivers’ modules (which are not their primary focus) because of the needs assessment where they were directly requested of their end-users. Several interviewees mentioned specifically involving end-users in quality of “expert by experience”. Lastly, end-users were involved in reading research proposals and thinking along.

One point of agreement in most of the interviewed participants was that, although caregivers are their primary target group, they cannot be considered as their “clients”, since they would not want caregivers to pay for the services themselves. In fact, some more financially strategic stakeholders were mentioned (especially commercial technologies). These stakeholders who were involved in order to settle financial agreements are municipality representatives, health insurers, clinicians, caregivers’ organizations and healthcare organizations.

Finally, several interviewees mentioned “coaches” or “therapists” as human barriers or facilitators. In cases of, for example, blended therapy, the attitude of a facilitator toward the technology was considered as a big predictor of its success.

*The professional is key. If he or she does not believe in this happening* (using the technology) *you as a client have to be really assertive in actually getting it going” – Case 3) Terug in balans (voor naasten) - lit: “back in balance (for loved ones)*.”

### 3.5. Value proposition

In business modeling, value propositions are the offerings of an organizations targeted to the needs of specific groups of customers (13). This family includes codes like “psychological values” and “practical values”. Accordingly, two main sets of values were identified in the interviews. On the one hand, values related to psychological wellbeing are identified. For example, the adaptive websites (PiB and MB) report the intention to deliver self-directed support in which caregivers can help themselves by learning a method to face new challenges, guided by the principal of self- management. Moreover, technologies like Mantelzorg Balans or Mantelzorg, offer validation for difficult emotions (such as anger), and built their module to provide a mirroring function, helping caregivers to reflect on themselves. In line with this value, several included cases (Mantelzorg, PiB, MB, MD, Goed Ernaast, Corsi Online, Me-We) aim at helping the caregivers with preventing burnout, reflecting on their role, keeping a balance between caregiving duties and self-care. On top of this, an educational function about specific illnesses or on how to provide care is another frequent element in these technologies.

Of the many values that were mentioned, two stood out as most recurring within different cases. Almost all interviewed participants reported that their technology aims at being a reliable and trustworthy source of information. That would mean simplifying the lives of caregivers who are in search of legal, governmental or illness-specific information, who may not be able to tell apart reliable and unreliable or unclear sources of information. Another “universal” value was the irreplaceability of certain elements of human interaction, which were not or should not be replaced using technology. In the pilot case of “A casa ma non da soli”, the group element is actually the main learning device, and the reduced number of sessions and it being online hindered the formation of a group entity.

The other set of mentioned values are the values grouped under the code “practical values”. These values have less to do with content elements of the technologies, and more with the user experience. Among these for example, several technologies have decided that their modules should be self-paced, and users should be able to browse them according to what they need most. Gratuity for end-users, user-centeredness, engagement, security, privacy, anonymity, and easiness to use were also recurring values. Other values concern the outcomes of using the modules and the actual behavioral change (e.g., reduction of burden, stress, hours of care etc.).

Interestingly, there seems to be a good match between the values (practical or psychological) that guided the included technologies’ design and the elements of content and user experience the technologies actually offer (Tables 3, 4).

**Table 3:** Overview of practical values.

Case (s)	Practical values
Mantelzorg	Engagement (Linked to perceived usefulness and its effect to effectivity)
Mantelzorg	User-centered design
Luna	Reduction of hours of care, stress (practical outcomes/behavioral changes) extended independent living, improving caregiver experience (caregiving from a distance
Luna; Mantelzorg balans; Terug in balans (voor naasten), Goed Ernaast,	Easy to use
Mantelzorg Balans; Goed Ernaast	Anonymity (no account needed)
Terug in balans (voor naasten)	Security
Goed Ernaast	Gratuity
Anziani e non solo; Terug in balans (voor naasten); Goed Ernaast (ma anche Pib-Mb)	Self-Paced

**Table 4:** Overview of psychological values.

Case(s)	Psychological value
A Casa Ma Non da Soli, Mantelzorg, Goed Ernaast (also in digestible amounts/chunks), Me-We, Corsi Online, Mantelsorg Balans	Reliable source of information
Partner in Balans; Luna	Self-Management
Partner in Balans	Positivity; hope
Mantelzorg Balans; Mantelzorg	Mirroring function
Mantelzorg Balans; Mantelzorg	validation of difficult emotions
Mantelzorg Balans, Luna, A casa ma non da soli	Irreplaceability of human contact
Mantelzorg	Engagement (linked to perceived usefulness and its effect of effectivity)
Mantelzorg, Goed Ernaast	Balance (boundaries/burnout awareness/prevention)
Mantelzorg	Education about emotions
Mantelzorg; Goed Ernaast; Anziani e non solo	Education about caregiving
Mantelzorg	Holistic view on people/caregivers
Partner in Balans	Inclusivity

### 3.6. Lessons learned on implementation

The last category of codes is called lessons learned. In this section, additional practical tips and insights, as well as important considerations that emerged during the interviews are described. First, it is important to remind the reader that all the included cases in the present work are implemented and available in real life settings. However, some of the included cases were developed in academic settings, while others were developed by commercial companies. Furthermore, while most of the technologies described are implemented in semi-private, insurance-based healthcare systems, few others are available in a public healthcare context.

Regardless of these distinctions, interviewees from academia agree that the financing, maintenance, and hosting matters need to be discussed upfront, especially for those technologies that run on research grants. Missing this step would result in an implementation failure and abandonment of the innovation, leaving behind great waste at many different levels (e.g., for the end-users, who would not be able to use the tool; all stakeholders who invested time and resources in the technology's uptake), and would force the process to start over in the future, if a similar need is identified.

*“And I would recommend actually doing that* (discussing financing*) early, like before you do your effectiveness trial. So we really get that information from everyone and people will tell you all the time when you try and when you're developing something and you want to talk to them about who is going to pay for it” – Partner in Balans*

Another common feeling among interviewees with an academic background is that they are often not equipped or knowledgeable enough to really make implementation related choices, because their expertise is different. Particularly, interviewees reported feeling overwhelmed because of the great number of implementation frameworks to choose from, yet still feeling they could make the wrong decision.

Conversely, interviewees from the market side, not only have specific implementation knowledge, but their job title is often “implementation-related”. However, they usually go for more market driven approaches, as opposed to frameworks which are developed within the academic settings. What they value in their frameworks of choice is concreteness, agility, easiness to use, applicability to real-life situations and customizability.

Designing and launching technologies in a commercial company is a very different experience from academic design. Several of the implementation specialists (from the industry) interviewed for this multiple case study report getting the request of designing caregivers’ modules directly from end-users (Therapieland, Transfore, Minddistrict), as an add-on to their existing products. In fact, caregivers are often not the primary target group for these companies. This kind of fast-paced, timely or “on demand” reaction to end-users’ needs was not found within the technologies developed in academia, at least within the present study.

Lastly, the influence of the Covid-19 pandemic on the uptake of technologies is one of the most agreed points amongst the interviewees, and it had a double effect. On the one hand, the Covid-19 pandemic speed up the process of digitalization, acting as a catalyst and an external motivator. In fact, in most cases digitalization was the only possible option. Furthermore, it brought along what one implementation specialist defined as “Corona Peak”, that is the growth of specific products demand. On the other hand, aspects of human contact or group cohesion are harder to achieve in online settings. This is not only true for the user experience side (as already mentioned in the value section), but also for the relationships with stakeholders. In fact, contact with relevant stakeholders, especially end-users for this very hard to reach target group (e.g., for co-design purposes) was hindered. Therefore, researchers had to adopt alternative ways or touchpoints to reach end-users. Nevertheless, implementation specialists suggest taking advantage of the momentum and being receptive to these kinds of external catalysts.

## 4. Discussion

The aim of the present paper was to analyze and compare available eMental health technologies for informal caregivers, considering their implementation approaches and business models. Two pilot interviews were conducted, and 8 cases were included. Three technologies were developed in academia and 5 in the industry setting. Key differences concerning efficacy testing and use of implementation frameworks were identified between eMental health technologies developed in academia and within the industry.

In line with previous literature (36), most technologies included in this case study were not evaluated for efficacy. The few exceptions were developed in academia and used rigorous evaluation methods (such as RCTs). Alternative testing methods encountered within the cases were small pilot rounds and usability testing. Conversely, technologies developed in the industry were not tested for efficacy at all, although in most cases end-users are involved in the design process to some extent. Although delivering evidence-based tools would be the gold standard, it could be argued that RCTs are not necessarily the best option given their high costs in terms of time and resources (37). For this reason, it is necessary to explore more viable alternatives to deliver evidence-based eMental health technologies for informal caregivers, by considering leaner methods of efficacy testing. Optimally, academia and industry should follow similar trajectories and adopt common standards.

Secondly, implementation experts from the industry have no experience with academic-driven implementation frameworks, and often end up creating their own or adopting more agile market-driven implementation approaches. More specifically, they generally agreed that academic implementation frameworks are often complicated, abstract, and difficult to translate into concrete steps. On the other hand, interviewees from academia were usually more familiar with academic-driven implementation frameworks but did not necessarily find them helpful or simple to use. This result is in line with Birken and colleagues (38), who pointed out a wide underuse, misuse, or superficial use of implementation frameworks. According to the authors, this would be at least partially related to the challenge of selecting from the many possibilities available. This sentiment is coherent with the interviewees’ responses within the context of this study, who reported feeling “inexperienced” and “afraid to go for the wrong frameworks” even after long consideration.

Although numerous implementation frameworks and theories exist, and implementation science is gaining popularity, academics and non-academics trying to implement new eHealth or eMental health technologies find themselves lacking practical guidelines. Only recently, a checklist eHealth implementation guideline was published by Cremers and colleagues (39). The scholars identified several domains and determinants that are important to keep in mind when developing eHealth, which are generally in line with the results of the present study. For example, financing and characteristics of the technologies are also found to be crucial elements of success. However, the checklist only aims at assessing “the determinants of successful eHealth intervention prior to the implementation of the eHealth program”. Therefore, a practical stepwise guideline is still missing. Implications from the present work expand on Cremers and colleagues (39) resulting in the first steps to build a practical and stepwise approach.

Generally, technology development in the industry side moves at a faster pace. In fact, experts from the industry side describe a short time frame between identification of the target group needs and the actual realization of the technologies. At times, these needs have been explicitly communicated to the companies by end-users, and companies were responsive to those needs. However, it seems that experts from the industry side might have different understandings of what implementation entails, with ideas of it being limited to the efforts that are necessary to get their clients (who often are not end-users), to purchase their products in licensing. In line with this view, they often opt for more market driven implementation guidelines.

Some touchpoints and similarities were also identified. First, both interviewees from academia and industry consider financial issues to be among the most relevant determinants for successful implementation. The most important lesson learned that was extracted from the interviews in this regard is that financing is a crucial aspect that needs to be discussed early on when designing a technology, since it greatly influences subsequent implementation decision making processes. This is especially true for technologies developed in academia since their financial situation tends to be more uncertain and reliant on research grants. Disregarding these aspects would result in abandonment of the innovation as soon as the project ends.

According with this line of thought, all technologies designed in an academic context included in the present study made more or less sophisticated arrangements to ensure continuity to their innovation. As a result, all of them can be found on the market and are available to use. Interviewees agree that neglecting to make arrangements for maintenance of the tools would automatically result in implementation failures and waste. This element is not only detrimental to the lifespan of a single technology but has greater impact as well. In fact, according to Kwasnicka and colleagues (37), if more eHealth technologies were successfully maintained, it would not be necessary to develop new ones from scratch, optimizing resources and time. Instead, existing tools could be adapted to new target groups or aims.

Furthermore, all interviewees agreed that involving stakeholders is an essential step to develop and implement their technologies. However, most cases did not describe adopting a systematic approach for stakeholder involvement. Specifically, no spontaneous reference was made to specific steps of stakeholder involvement such as stakeholder identification or stakeholder analyses (13). A more systematic approach was only adopted by Partner in Balans, since the researchers involved different stakeholders in an interview study with the aim of structuring the business model and implementation strategy of their innovation (25).

Structuring a business model is a good strategy to possibly prevent implementation issues and its added value was recognized from most interviewers both with academic and industry background. Business modelling requires reflection about key implementation elements such as: relevant stakeholders of an innovation, what are the values, how does the revenue stream flow and who is going to pay for the tool. Also, interviewees who did not structure a proper business model for their technology reported regretting the choice and declared they would use it in the future.

The present work is not short of limitations, including the representativity of the sample, the interpretation of results and common limitations of qualitative research. First, only examples of European eMental health technologies are included. Also, these technologies were selected through snowball sampling and word-of-mouth, making the pool more susceptible to the connections of the researchers involved. Moreover, context and healthcare-type specific considerations need to be kept in mind when interpreting these results. For example, technologies which would be successful in a semi-private healthcare system, would probably not be sustainable in a public healthcare system. Also, since the Netherlands reported the highest technology literacy scores in Europe in 2021 (40). Therefore, local levels of digital literacy need to be considered as predictors of success, in case of sophisticated eHealth technologies. Finally, well-known limitations of qualitative research methods should be acknowledged. For example, social desirability might have influenced the interviewee responses, and qualitative coding and analyses are susceptible to researchers’ biases.

## 5. Conclusion

The main aim of the present work was to describe and compare eMental health technologies for informal caregivers developed within academia and industry, while extracting lessons learned for their implementation. Mainly, the work sheds light on existing differences in terms of efficacy testing and use and choice of frameworks. Also, similarities in terms of recognized barriers, such as financing, and possible ways to overcome them, such as structuring a business model were uncovered. We hereby propose three points for reflection and future research directions, building on our main results. First, next steps in research could be focused on getting these differently working environments joining forces to combine their strengths. For example, by exploring more agile ways of delivering evidence-based content or providing testimonies to the combination or experience with academic frameworks and in real life settings. Moreover, it would be interesting to explore how the adaptation of existing frameworks, or the creation of new ones occurs within these organizations, both from a process and a content side. In this logic, implementation frameworks would serve as supportive guidelines, rather than unmodifiable stepwise recipes, thus (i) empowering the implementation team to a more active role in implementation and (ii) reducing the pressure and confusion of picking the right framework. Finally, a new stakeholder group could be considered in the design phase. As often mentioned during these interviews, developers often outsource tasks to external companies (e.g., researchers often outsource software related elements of design to tech-companies) a similar service could be provided by “implementation experts”. In other words, professionals could provide implementation-specific consultancy for researchers or developers within the industry to guide their decision making.

## Data Availability

The datasets presented in this article are not readily available because it is not possible to fully anonymize the dataset. Requests to access the datasets should be directed to SB, s.bastoni@utwente.nl.
