# Supplementary material for: Implementation of eMental health technologies for informal caregivers: A multiple case study
Source: Front Digit Health. 2023 Mar 24;5:1130866. doi: 10.3389/fdgth.2023.1130866 (PMC10081674; doi:10.3389/fdgth.2023.1130866)
Supplement: Supplementary file 1 [file Datasheet1.docx]

**Interview guide for semi-structured interviews with Implementation experts of eHealth technologies interventions to improve mental wellbeing of informal caregivers and caregiver experience**

Time for mutual introductions

Let’s start with a couple of general questions:

1. How would you describe your innovation in a couple of sentences? What are the most important things to say about it?
2. Tell me about the creative process. How did the idea come to life? (Specific need to address and theoretical model you followed, ties with academia, tested for efficacy?)
3. What would you say are the core values of your innovation? (Value based design, value specification, value proposition)

Ok, thank you. Now, I would like to ask you some questions about implementation

1. Tell me about your organization (How big it is, structure, complexity level)
2. What is your role in the organization and how were you involved in its implementation? Who else is involved in implementation (formally or informally)?
3. In which stage it is? How do you measure it?
4. What kind of planning was done before the technology was implemented?
5. In what ways was a framework or theory used to guide implementation? (How did you choose one to use?)
6. Did Covid influence with your implementation? (How?)

Great, now the idea is that I will present to you several general themes. I will ask you to reflect on how these themes influenced the implementation of the technology.

1. Attributes of the technology itself? Hardware? How do you deal with updates and technical support?
2. Organizational context? (Relationships with local organizations, specific implementation knowledge, etc)
3. Wider context? (Socio-political, economical, healthcare,)
4. Stakeholders? (Who are they, how they were identified, what is the specificity of dealing with caregivers, were they involved in design/distribution, other phases, what was the added value, describe the process, what challenges did you encounter?)

As a last question, can you think of any other technologies that I might want to involve in my case study? Is there someone else you think I would benefit from talking to?

Thank you, the interview is finished. As part as a short follow up, I will fill in a Business Model Canvas regarding your intervention and share it with you, would you be willing to complete it?
